# A Prospective Analysis of Surgical Site Infections in the Neck of Femur Fractures in a Trauma Unit: A Six-Year Analysis From 2019 to 2024

**DOI:** 10.7759/cureus.83545

**Published:** 2025-05-05

**Authors:** Francesca Solari, Claire Joyner, Kumar Dash, Shady Hanna, Kelly Porter, Victoria Williams, Aso Mohammed

**Affiliations:** 1 Trauma and Orthopaedics, University Hospital of Wales, Cardiff, GBR; 2 Trauma and Orthopaedics, Prince Charles Hospital, Merthyr Tydfil, GBR; 3 Trauma and Orthopaedics, The Grange University Hospital, Cwmbran, GBR; 4 Trauma and Orthopaedics, Morriston Hospital, Swansea, GBR

**Keywords:** deep incisional ssi, neck of femur fracture (nof), neck of femur fractures, superficial ssi, surgical site infection (ssi)

## Abstract

Background

Surgical site infections (SSIs) following neck of femur fracture surgery are associated with increased morbidity, mortality, and healthcare costs. Identifying risk factors for SSIs is essential for improving patient outcomes.

Methods

We conducted a prospective cohort study at Morriston Hospital, Swansea, United Kingdom, analysing neck of femur fracture surgeries performed between 2019 and 2024. Data were collected on ASA (American Society of Anesthesiologists) grade, Abbreviated Mental Test (AMT) score, duration of operation, time to theatre, skin closure method, grade of surgeon, and operation type. Statistical analysis was performed using univariate (Chi-squared and Mann-Whitney U) and multivariate logistic regression analyses.

Results

A total of 1,737 cases were included. The overall SSI rate was 4.3%, with a reduction from 9.43% in 2019 to 1.93% in 2024. Skin closure method influenced infection rates, with a notable reduction in SSIs following the transition from clips (88.3% in 2019 to 5.8% in 2024) to Monocryl (11.3% to 93.3%). On univariate analysis, significant associations were found between SSI incidence and closure method (p = 0.022), time to theatre (p < 0.0001), and operative duration (p < 0.0001). On multivariate analysis, higher AMT scores were found to be protective against infection (p < 0.0001 for AMT 8, and p = 0.023 for AMT 9). Additionally, hemiarthroplasty and intramedullary nailing were protective against infection, both with p < 0.0001.

Conclusion

Findings suggest that the skin closure method plays a role in SSI risk following neck of femur fracture surgery. Further prospective studies are needed to validate these findings and explore additional risk factors. This study contributes to optimising surgical techniques and improving patient outcomes.

## Introduction

Post-operative wound infections remain a significant concern in trauma surgery, particularly in patients undergoing operative management for neck of femur fractures. Neck of femur fractures, commonly seen in the elderly population, are associated with high morbidity and mortality rates. These fractures usually require surgical intervention, and the subsequent risk of complications including surgical site infections (SSIs) can significantly impact recovery, prolong hospital stay, and increase mortality [1].

SSIs following neck of femur fracture surgery are influenced by multiple factors, including patient co-morbidities, the type of wound closure material, and overall infection control practices. Surgical wound closure plays a critical role in the prevention of SSIs, with various methods including the use of absorbable sutures or non-absorbable materials like surgical clips showing different outcomes in terms of infection rates [2]. Absorbable sutures, which dissolve naturally over time, have been suggested to minimise the risk of infection by reducing the need for a second procedure to remove them. However, surgical clips, which are easier and quicker to apply, remain widely used in orthopaedic surgeries [3]. The debate around the optimal wound closure method for minimising SSIs is ongoing.

The American Society of Anesthesiologists (ASA) classification system, which assesses the physical status of patients prior to surgery, has been shown to correlate with postoperative complications, including SSIs [4]. Similarly, cognitive impairment, measured using tools like the Abbreviated Mental Test (AMT) score, may influence a patient’s ability to follow postoperative care instructions, potentially increasing the risk of infection [5].

This prospective study was conducted at Morriston Hospital, a trauma unit in Wales, utilising data from the Theatre Operating Management System (TOMS) and the Welsh Clinical Portal. It aimed to compare the incidence of SSIs in neck of femur fracture patients and determine whether closure type affects this, alongside other variables such as time to theatre, operation duration, operation type, ASA, AMT, and operating surgeon grade. By analysing these variables within six years of data, this study seeks to contribute to the ongoing discussion regarding the optimal approach to wound closure in this vulnerable patient population, with the ultimate goal of reducing postoperative complications and improving patient outcomes.

## Materials and methods

Study design

This study was a prospective analysis of SSIs in patients undergoing surgery for neck of femur fractures at Morriston Hospital, Swansea Bay University Health Board. Data collection was conducted over six consecutive years. Initially, data were collected from 160 consecutive patients who underwent surgery in 2019 and 2020. Subsequently, all eligible patients treated over six-month intervals from 2021 to 2024 were included. Adjustments in data collection reflected changes in personnel availability and adaptations following the COVID-19 pandemic, resulting in a more robust process and an extended collection period. 

Patient selection

Patients were included if they underwent surgery for a neck of femur fracture or periprosthetic hip fracture at Morriston Hospital during the specified timeframes. Patients were excluded if they did not undergo operative intervention. Patients were identified from daily inpatient handover lists. Data on operation type, surgeon grade, closure type, time to surgery, and operative duration were extracted from Morriston Hospital’s theatre database (TOMS). Information regarding the presence or absence of an SSI was obtained from the national electronic medical record system (Welsh Clinical Portal).

Definitions

SSIs were defined as an infection occurring in a part of the body that had been operated on [6]. SSIs were further classified as (I) superficial - infection occurring within the skin within 30 days of surgery, and (II) deep incisional - infection involving deep tissues occurring within 30 days of surgery or up to one year postoperatively, with purulent discharge and/or a positive culture result, as per the Musculoskeletal Infection Society criteria [7]. In this study, confirmation of an SSI required a positive culture result from either deep or superficial wound specimens.

Statistical analysis

Binary outcomes (infection vs. no infection) were analysed using Mann-Whitney U and Chi-squared tests to assess associations between infection and continuous or categorical variables, respectively. Mann-Whitney U was utilised as the continuous data was not parametric in the analysis. Multivariate logistic regression was then performed to identify independent predictors of SSI. A p-value <0.05 was considered statistically significant. Analyses were conducted using XLStat (version 2024.4.0; Lumivero, Denver, CO, USA).

Ethical considerations

This study is a service evaluation conducted within our trauma unit, assessing SSIs in neck of femur fractures over a six-year period. As per the guidance of the Health Research Authority (HRA) and local governance policies, service evaluations aim to assess current practice and do not involve changes to patient care, randomisation, or additional interventions beyond standard treatment. Therefore, ethical approval was not required for this study. Patient data were handled in accordance with institutional guidelines, ensuring confidentiality and compliance with data protection regulations. No identifiable patient information is included in our submission.

## Results

Descriptive statistics

Table 1 summarises the descriptive analysis of the dataset collected over six years. A total of 1,737 cases were analysed, with 74 (4.3%) developing an SSI. The mean operation duration was 84.6 minutes, and the mean time to theatre was 49.8 hours. Infection rates were highest in 2019 (n = 15, or 9.43%) and 2022 (n = 15, or 4.24%), with the lowest rate in 2024 (n = 9, or 1.93%). Overall, 74 (4.3%) of cases developed an infection.

**Table 1:** Descriptive breakdown of all categories analysed DHS, Dynamic Hip Screw; IM Nail, Intramedullary Nail; ORIF, Open Reduction and Internal Fixation; THR, Total Hip Replacement; SHO, Senior House Officer; ASA, American Society of Anesthesiologists; AMT, Abbreviated Mental Test

	Categories	Frequency per Category (N)	Relative Frequency per Category (%)
Year	2019	159	9
	2020	159	9
	2021	288	17
	2022	354	20
	2023	311	18
	2024	466	27
Infection	Infection	74	4
	No Infection	1663	96
Operation	Cannulated Screws	2	0
	DHS	414	24
	Girdlestone	8	0
	Hemiarthroplasty	758	44
	IM Nail	366	21
	ORIF Femur	60	3
	Revision THR	4	0
	THR	122	7
Grade	Consultant	825	48
	Registrar	841	49
	SHO	59	3
	SHO	2	0
Closure	Clips	213	13
	Ethilon	13	1
	Monocryl	1461	86
	Vicryl Rapide	7	0
ASA	0	11	1
	1	13	1
	2	289	17
	3	914	53
	4	497	29
AMT	0	330	20
	1	47	3
	2	49	3
	3	50	3
	4	49	3
	5	47	3
	6	44	3
	7	81	5
	8	159	10
	9	219	13
	10	583	35

Hemiarthroplasty was the most common procedure, performed in 49.9% of cases (n = 758), followed by intramedullary nailing (25.5%, or n = 366) and dynamic hip screw fixation (16.4%, or n = 414). Consultants performed 49.5% of operations (n = 825), registrars performed 45.8% (n = 841), and SHOs (Senior House Officers) performed 4.7% (n = 59). The most frequent ASA grade was 3 (56.1%, or n = 914), followed by ASA 4 (29.1%, or n = 497). The most common AMT score was 10 (31.7%, or n = 583), followed by score 9 (14.3%, or n = 219) and score 8 (13.6%, or n = 159). One notable trend across the years was the shift from clips to Monocryl for wound closure. In 2019, clips were used in 88.3% (n = 141) of cases, while Monocryl was used in only 11.3% (n = 18). By 2024, Monocryl usage had increased significantly to 93.3% (n = 435), with clips falling to 5.8% (n = 25). This switch in closure method was the only major change observed across the descriptive statistics. Table 2 demonstrates this, showing the breakdown of closure types per year and associated infection rates.

**Table 2:** Showing frequencies of cases, infections, and closure types per year

	2019	2020	2021	2022	2023	2024
Number of Neck of Femur Fractures	159	159	288	354	311	466
Number of Infections (%)	15 (9.43)	7 (4.40)	12 (4.16)	15 (4.24)	17 (5.47)	9 (1.93)
Closure With Clips (%)	141 (88.3)	9 (5.6)	29 (10.0)	22 (6.3)	8 (2.6)	27 (5.8)
Closure With Monocryl (%)	18 (11.3)	133 (83.7)	249 (86.5)	329 (92.9)	303 (97.4)	435 (93.3)
Other Closure (%)	1 (0.6)	17 (10.7)	10 (3.5)	3 (0.8)	0 (0)	4 (0.9)

Univariate analysis

Table 3 demonstrates the univariate analysis results for both continuous and categorical variable groups to assess whether there was an association between these variables and the presence of an infection. Chi-squared tests identified significant associations between infection rates and wound closure type (p = 0.022), as well as time to theatre (p < 0.0001) and operative time (p < 0.0001). No significant associations were found for surgeon grade (p = 0.397), ASA score (p = 0.517), AMT score (p = 0.309), or operation type (p = 0.069). Mann-Whitney U tests confirmed that infected cases had significantly longer time to theatre (p < 0.0001) and operative time (p < 0.0001).

**Table 3:** Univariate analysis results Bold values indicate statistically significant results, defined as p < 0.05. ASA, American Society of Anesthesiologists; AMT, Abbreviated Mental Test

Variable	Data Type	Test	Test Statistic	p-value
Operative Time	Continuous	Mann-Witney U	0	<0.0001
Time to Theatre	Continuous	Mann-Witney U	24557.500	<0.0001
AMT	Categorical	Chi-squared	11.527	0.309
ASA	Categorical	Chi-squared	2.827	0.517
Closure	Categorical	Chi-squared	12.947	0.022
Operation Type	Categorical	Chi-squared	16.080	0.069
Surgeon Grade	Categorical	Chi-squared	2.274	0.397

Multivariate analysis

Logistic regression analysis identified independent predictors of SSI. Operative time was inversely associated with infection risk (OR = 0.92, 95% CI: 0.86-0.98, p = 0.019), suggesting that longer procedures were linked to a lower likelihood of infection. Time to theatre did not reach statistical significance in the multivariable model (p = 0.095).

Compared to total hip replacement (THR), both hemiarthroplasty (OR = 0.75, p < 0.001) and intramedullary nailing (OR = 0.73, p < 0.001) were associated with a significantly lower risk of infection. Neither Monocryl nor clips showed a statistically significant association with infection, though large standard errors suggest instability in these estimates.

Lower cognitive function, as measured by AMT score, was associated with a higher risk of infection. AMT-8 (OR = 0.83, p < 0.001) and AMT-9 (OR = 0.92, p = 0.023) were both linked to lower odds of infection (Table 4).

**Table 4:** Logistic regression analysis results Bold values indicate statistically significant results, defined as p < 0.05. DHS, Dynamic Hip Screw; IM Nail, Intramedullary Nail; ORIF, Open Reduction and Internal Fixation; THR, Total Hip Replacement; SHO, Senior House Officer; ASA, American Society of Anesthesiologists; AMT, Abbreviated Mental Test

Variable	Coefficient	Standard Error	Wald Chi-Square	p-value	Odds Ratio (OR)
Operation Duration	-0.005	0.002	5.525	0.019	0.995
Time to Theatre (Hours)	0.002	0.001	2.791	0.095	1.002
Operation - Cannulated Screws	17.779	21107.55	0	0.999	-
Operation - DHS	0.299	0.318	0.888	0.346	1.349
Operation - Girdlestone	18.439	7791.049	0	0.998	-
Operation - Hemiarthroplasty	-1.038	0.284	13.36	<0.0001	0.354
Operation - IM Nail	-1.289	0.299	18.638	<0.0001	0.276
Operation - ORIF Femur	0.233	0.498	0.219	0.64	1.262
Operation - Revision THR	18.405	11724.69	0	0.999	-
Operation - THR	0	0	-	-	-
Grade - Consultant	-18.845	14561.94	0	0.999	0.993
Grade - Registrar	-18.66	14561.94	0	0.999	1.124
Grade - SHO	-19.117	14561.94	0	0.999	1.112
Closure - Clips	-20.243	7723.435	0	0.998	0.997
Closure - Ethilon	0.421	9519.704	0	0.999	0.989
Closure - Monocryl	-19.206	7723.435	0	0.998	0.996
Closure - Vicryl Rapide	0	0	-	-	-
ASA - 0	19.211	7778.564	0	0.998	-
ASA - 1	19.402	5575.045	0	0.997	-
ASA - 2	0.262	0.198	1.755	0.185	1.299
ASA - 3	-0.017	0.137	0.016	0.899	0.983
ASA - 4	0	0	-	-	-
AMT - 0	-0.421	0.167	6.388	0.011	0.656
AMT - 1	18.632	2990.194	0	0.995	-
AMT - 2	18.898	2815.966	0	0.995	-
AMT - 3	-0.764	0.305	6.274	0.012	0.466
AMT - 4	0.615	0.399	2.379	0.123	1.85
AMT - 5	-0.197	0.328	0.359	0.549	0.822
AMT - 6	-0.548	0.346	2.5	0.114	0.578
AMT - 7	0.445	0.302	2.166	0.141	1.56
AMT - 8	-0.965	0.199	23.55	<0.0001	0.381
AMT - 9	-0.417	0.183	5.174	0.023	0.659
AMT - 10	0	0	-	-	-

## Discussion

The results of this study contribute valuable insights into the factors influencing the incidence of SSIs following surgery for neck of femur fractures. With a total infection rate of 4.3% across 1,737 cases, the findings highlight the significance of wound closure techniques, operative time, and patient-related factors, such as cognitive function, as predictors of infection risk.

The shift in wound closure methods observed over the six years of the study is noteworthy, with an increase in the use of absorbable sutures (Monocryl) and a corresponding decrease in the use of surgical clips. This change aligns with growing evidence suggesting that absorbable sutures, such as Monocryl, may reduce the risk of SSIs compared to non-absorbable materials like clips. Previous studies have demonstrated that absorbable sutures, by eliminating the need for removal and reducing foreign body presence, can lower infection rates [8,9]. In our study, the association between wound closure type and infection risk was statistically significant (p = 0.022) on a univariate analysis. After including other factors in the multivariate analysis, this significance was lost for association with the closure method and infection. However, the large standard error seen in closure could be contributory; the observed trend suggests that wound closure methods deserve further investigation in larger, more controlled studies. Considering the significant shift from 2019, from clips to Monocryl, and the disparity in numbers - overall, 213 clips compared to 1,461 Monocryl closures - could be contributory to the large standard error and the lack of a clear association. A recently published international Delphi study on wound closure and incision management in hip arthroplasty has found consensus that sutures for skin closure may lower the risk of superficial SSIs compared to staples [10].

The analysis also revealed significant relationships between infection rates and operative time and time to theatre, consistent with previous literature indicating that prolonged surgery and delayed intervention increase the risk of infection [11]. The finding that longer operative time was inversely associated with infection risk (OR = 0.92, p = 0.019) is contrary to what might be expected, where one would anticipate that longer procedures would increase the risk of infection. This finding may be explained by differences in the types of procedures performed in longer operations, such as the more complex surgeries that may be associated with better infection control practices, or different patient demographics that influence infection susceptibility. These operations may have been more consultant-led and may have necessitated further intraoperative antibiotic doses as prophylaxis. Further research could explore whether certain procedures, operative complexity, or surgical team factors, including overall theatre traffic and number of scrubbed team members, are associated with infection rates.

Time to theatre was also significantly associated with infection risk in the univariate analysis, supporting the hypothesis that delayed surgery can lead to a higher incidence of SSIs. However, this factor did not reach statistical significance in the multivariate model (p = 0.095), suggesting that other variables, such as the type of surgery or the patient’s cognitive status, may mediate this relationship. The role of time to theatre in infection risk is well-documented, with several studies highlighting that earlier surgical intervention is critical in reducing infection rates, particularly in trauma cases [12]. It is also worth understanding that the delayed surgery group may not always be homogeneous. Subgroup analysis in the WHITE multicentre study on performance-based remuneration showed that the “medical delay” (clinical decision to delay to correct a modifiable pre-surgery risk) group performs worse than the “administrative delay” (lack of theatre time or space) group in terms of mortality and EQ-5D scores [13].

Cognitive function, as measured by the AMT score, was another important factor associated with infection risk in the multivariate analysis. Lower cognitive function, which is often seen in elderly patients with neck of femur fractures, was linked to an increased likelihood of SSIs. This finding suggests that patients with cognitive impairments may have a reduced ability to adhere to postoperative care instructions, increasing their vulnerability to infection. Previous research has identified cognitive impairment as a risk factor for poor postoperative outcomes, including infections, due to a compromised ability to follow medical recommendations [14]. When compared to people without dementia, a diagnosis of dementia is associated with double the rate of urinary incontinence and triple the rate of faecal incontinence [15]. This can be due to either deterioration in physical control of voiding or behavioural/psychological issues manifesting as inappropriate voiding behaviours. Hence, the need for a good skin closure method and wound protection becomes important in our neck of femur fracture surgery patients to prevent secondary contamination and infection. These findings further emphasise the need for tailored postoperative care strategies for patients with cognitive impairments, such as additional support or more frequent monitoring.

Surgeon grade, ASA score, and operation type did not show significant associations with infection risk in the multivariate analysis. While it is well-established that surgeon experience and patient co-morbidities can influence surgical outcomes [16], the lack of significant findings here may be attributed to the homogeneity of the surgical team or the fact that the ASA score and operation type were not as strongly correlated with infection risk as other factors, such as cognitive function or wound closure method.

Interestingly, the infection rates varied significantly across the years, with a higher rate observed in 2019 and 2022, and a notable decline in 2024. This variation could reflect improvements in surgical protocols, infection control measures, or changes in the patient population over time. The increase in subcuticular absorbable suture (Monocryl) usage instead of skin clips, and the decreasing infection rate in 2024, suggest that changes in wound closure methods, along with other possible improvements in care, may have contributed to the reduction in infection rates. While the current study cannot establish a causal relationship between subcuticular absorbable suture (Monocryl) usage and reduced infection rates, the observed trends warrant further investigation into the effectiveness of specific wound closure methods in preventing SSIs. The change in 2019 from the majority of skin clips to subcuticular absorbable suture (Monocryl) closure - a shift from 88.3% clips in 2019 to 83.7% subcuticular in 2020 - was the single change in protocol over this time period. The number of infections remained low, so this makes analysis challenging, even with a reduction in infection from 9.43% in 2019 to 1.93% in 2024.

Despite the findings, there are several inherent limitations with the current study. This was a single-centre assessment, potentially limiting the generalisability of the results. Local practice, patient cohort, and protocols may not be representative in other centres, affecting the external validity of the findings. As this was a prospective analysis, there was no a priori power calculation governing the sample size required to detect statistical significance, raising the possibility that this may be underpowered, especially when considering small effect sizes. In addition, some results demonstrated large standard errors, indicating a high degree of uncertainty in the estimates. It is also key to point out that, after the first year of data collection, there was a significant change in practice, with the majority of closures moving from clips to Monocryl. With no data before this, the numbers remain relatively small for clip closures. With this, the ability to ascertain long-term trends is challenging, and understanding that small changes in infection rates could have a disproportionate impact on the statistical analysis. 

## Conclusions

In conclusion, this study provides valuable evidence regarding the factors influencing the incidence of SSIs in neck of femur fracture surgeries. The findings suggest that, while the type of wound closure, particularly the use of absorbable sutures, is an important consideration, other factors - such as operative time, time to theatre, and cognitive function - also play significant roles in infection risk. Although no single factor was identified as the definitive cause of infection, the cumulative evidence highlights the importance of a multifactorial approach to infection prevention, including optimising surgical technique, timely intervention, and individualised care for patients with cognitive impairments. Future research, particularly prospective randomised trials, will be essential to further elucidate the relationship between wound closure methods and infection rates and to refine strategies for minimising postoperative complications in this high-risk population.
